# A novel compound heterozygous SPG7 variant is associated with progressive spastic ataxia and persecutory delusions found in Chinese patients: two case reports

**DOI:** 10.1186/s12883-022-02706-1

**Published:** 2022-05-30

**Authors:** Shan Wang, Yaye Wang, Yue Wu, Jinru Zhang, Weilin Zhang, Chang Li, Xueqin Song

**Affiliations:** 1 Department of Neurology, The Second Hospital of Hebei Medical University, Shijiazhuang, 050000 Hebei China; 2 Neurological Laboratory of Hebei Province, Shijiazhuang, Hebei 050000 People’s Republic of China

**Keywords:** SPG7, HSP, Novel variant, Cognitive impairment, Spastic ataxia, Psychosis

## Abstract

**Background:**

Hereditary spastic paraplegia 7 (SPG7) is one of the subtypes of autosomal-recessive hereditary spastic paraplegia, which is a clinically heterogeneous neurodegenerative disorder. SPG7 often displays a complicated phenotype, including optic atrophy, ophthalmoparesis, and impaired emotional communication. In the Chinese population, sporadic cases of SPG7 variant-associated spastic ataxia are rarely reported.

**Case presentation:**

We carefully analysed the clinical features, imaging and genetic tests of two sporadic patients with SPG7, both from the Hebei region of China. One patient presented with progressive bilateral lower limb weakness, spastic-ataxia and no cognitive impairment. Brain MRI revealed mild cerebellar atrophy. Genetic analysis revealed c.1150_1151insCTAC (p.G384Afs*13) frameshift variant and exon1-3 heterozygous deletion. The other patient presented with progressive bilateral lower limb weakness, ataxia, dysarthria and a mild psychosis associated with persecutory delusions, which drew almost no attention, in addition to mild cognitive impairments characterized by a decrease in verbal memory and executive function. Genetic analysis identified two heterozygous variants in the SPG7 gene: c.1150_1151insCTAC (p.G384Afs*13) and c.1496delC (p.Q500Sfs*13).

**Conclusions:**

The c.1496delC (p.Q500Sfs*13) variant in exon 11 has not been reported before. The c.1150_1151insCTAC variant is speculated to be a hotspot variant in the Chinese population. Patients with SPG7 may have cognitive impairments and psychosis, displaying specific characteristics, which should be of concern.

**Supplementary Information:**

The online version contains supplementary material available at 10.1186/s12883-022-02706-1.

## Background

Hereditary spastic paraplegia 7 (SPG7) is one of the subtypes of autosomal-recessive hereditary spastic paraplegia (HSP), which is a clinically heterogeneous neurodegenerative disorder [1]. This disorder normally displays complex phenotypes, in addition to the common spastic-ataxia mimicking spinocerebellar ataxia or multiple system atrophy type C, including optic atrophy, cognitive/behavioural impairments, amyotrophy, ophthalmoparesis and peripheral neuropathy, which is easily misdiagnosed clinically [2]. In the Chinese population, sporadic cases of SPG7 variant-associated spastic paraplegia are rarely reported. Here, we summarized the genetic and phenotypic characteristics of the disease by analysing two families with SPG7, and we identified a novel SPG7 variant and a phenotype of mild psychosis associated with persecutory delusions rarely described in the literature, further strengthening clinicians' awareness of the disease for early genetic counselling.

### Case presentation

**Fig. 1:**
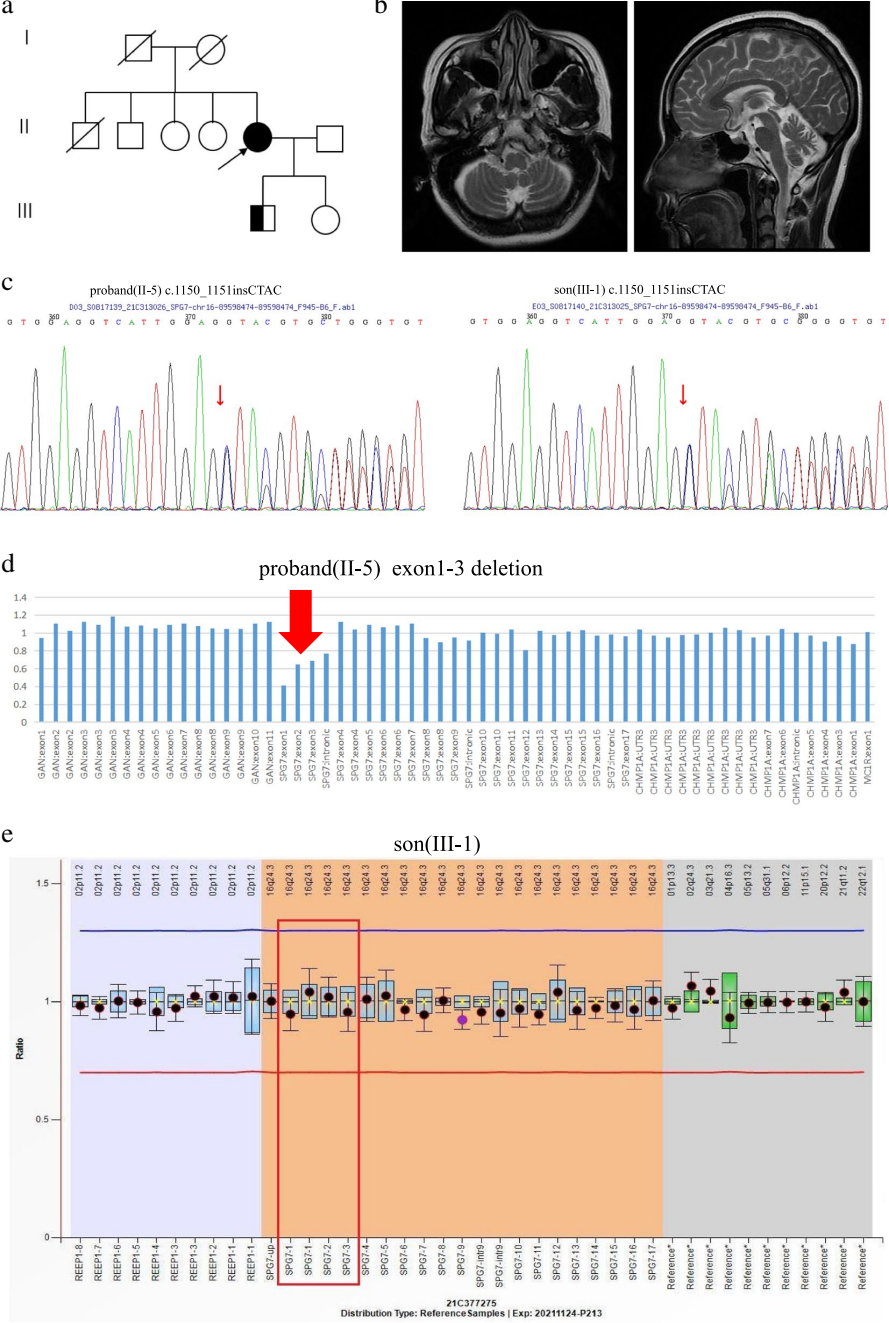
Imaging and genetic results of family 1. **a** Pedigree of SPG7 family 1. The proband is indicated by an arrow. **b** Axial and sagittal T2-weighted brain MRI showing cerebellar atrophy. **c** Sanger DNA sequencing chromatogram demonstrating the heterozygosity for the c.1150_1151insCTAC variant in the proband and her son. **d** Exon 1–3 deletion detected by NGS CapCNV analysis of proband. **e** Genetic analysis of hereditary spastic paraplegia using multiplex ligation-dependent probe amplification(MLPA) on the son of the proband showed no copy number abnormalities in the exons of the relevant genes. ( It is generally recognized that the fluorescence signal intensity between 0.7–1.3 is normal, that is, between the red line and the blue line.)

The first proband (Fig. 1a, family 1) was a 48-year-old female who presented mainly with ataxia, proximal weakness of both lower limbs and a tendency to fall (Table 1) for 7 years. Initially, the patient was able to walk, but not in a straight line. Three years ago, the patient’s condition gradually worsened. She needed help climbing stairs and was easy to fall. The patient denied any family history of similar symptoms. Physical examination: both proximal lower limbs’ muscle strength, grade 4; bilateral finger-nose and heel-knee-shin tests were less accurate; positive bilateral Babinski’s sign; and positive Romberg sign with both open and closed eyes. The cranial nerves and sensory system examinations were normal. Meningeal irritation sign was negative. Brain MRI revealed mild cerebellar atrophy (Fig. 1b). Cervical and thoracic spinal cord MRI showed no abnormality. Electromyography did not suggest myogenic or neurogenic injury. The Scale for the Assessment and Rating of Ataxia (SARA) score was 9 (gait 2, standing 2, sitting 0, poor dysarthria 0, finger tracking test 1, finger-nose test 1, rapid alternation test 1, heel-knee-shin test 2). Cognitive ability screening showed no decline (Mini-mental State Examination, MMSE 30/30, Montreal Cognitive Assessment, MoCA 29/30).

**Table 1:** Clinical features of patients with SPG7 mutations

Test	Patient II-5	Patient II-3
Sex	F	M
Age at onset(years)	41	41
Disease duration (years)	7	15
Disability at last visit^a^	1	1
Initial symptoms	Ataxia	Ataxia, Dysarthria, Cognitive impairment, Psychosis
Muscle weakness (MRC scale): grade 0–5	Both proximal lower limbs 4 Other body strength 5	Both lower limbs 4 Other body strength 5
Tendon reflex	Bilateral biceps tendon reflexes + + + Bilateral knee tendon reflexes + +	+ +
Muscle tension	Normal	Hypertonia
SARA scale	9	8
Sensory nervous system and meningeal irritation sign	Normal	Normal
Babinski’s sign	Positive	Positive
Romberg sign	Open eyes + Close eyes +	Open eyes - Close eyes -
Cognitive Functioning Scale	MMSE 30 MoCA 29	MMSE 27 MoCA 22
Nerve conduction velocity	Normal	Normal
Needle EMG	Normal	Normal
Nucleotide change	c.1150_1151insCTAC exon1-3 del	c.1150_1151insCTAC c.1496delC

Abbreviations: *M* male, *F* female, *SARA* Ataxia Rating Scale, *MMSE* Mini-mental State Examination, *MoCA* Montreal Cognitive Assessment

^a^ Disability scale: 0 = no gait difficulties; 1 = disease onset as defined by onset of gait difficulties; 2 = loss of independent gait, as defined by permanent use of a walking aid or reliance on a supporting arm; 3 = confinement to wheelchair; 4 = death

Genetic testing revealed one heterozygous exon 1–3 deletion and one heterozygous c.1150_1151insCTAC variant (the Ensembl transcript ID with the NM number: ENST00000268704, NM_003119) in the SPG7 gene (Fig. 1c, d, e). Her 26-year-old son was found to be heterozygous for the SPG7 c.1150_1151insCTAC variant but did not reveal any subclinical signs or symptoms.

**Fig. 2:**
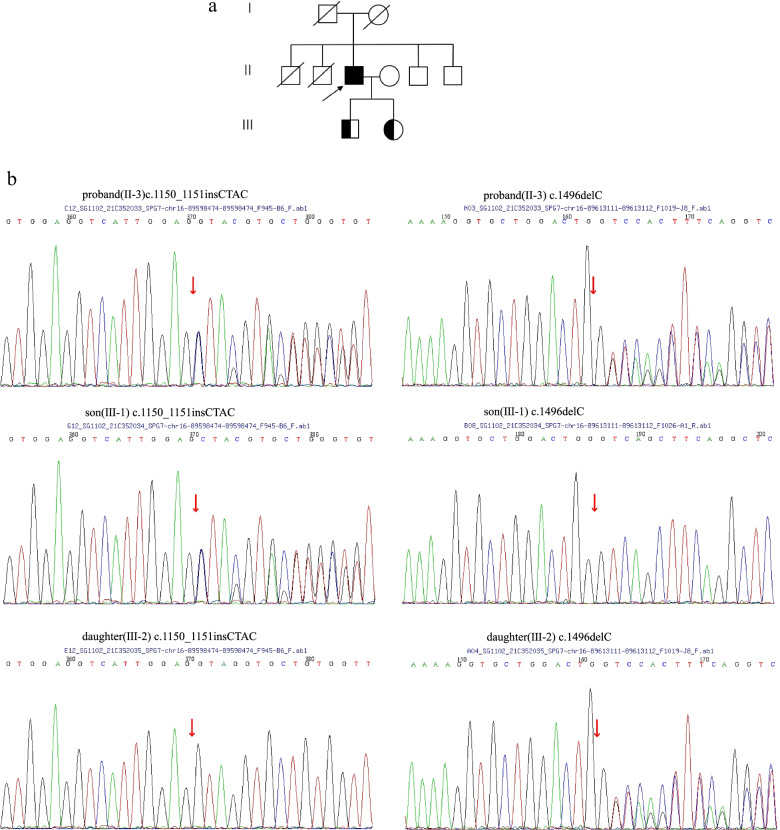
Genetic results of family 2. **a** Pedigree of SPG7 family 2. The proband is indicated by an arrow. **b** Sanger DNA sequencing chromatogram demonstrating the heterozygosity for the c.1150_1151insCTAC variant in the proband and his son and the c.1496delC heterozygous variant in the proband and his daughter

The second proband (Fig. 2a, family 2) was a 56-year-old male with 12 years of education who presented with bilateral lower limb weakness, unsteady walking, dysarthria and cognitive impairment (mainly impaired recent memory). The patient denied a family history of neurological disease. Examination showed grade 4 muscle strength of lower limbs, bilateral normal finger-nose test, bilateral inaccurate heel-knee-shin test, positive Babinski’s sign and scissor gait (Table 1). The cranial nerves and sensory system examinations were normal. Romberg’s sign was negative. Brain CT and craniocervical CTA showed cerebral arteriosclerosis without significant cerebellar atrophy (MRI was contraindicated due to the presence of a cardiac stent for coronary artery disease). The electromyogram showed no abnormal changes. Neuropsychological testing (Additional file 1, Table S1) revealed that the patient suffered mild cognitive impairment, including impaired verbal recall, reduced object naming and executive dysfunction (a poor time score in the Stroop test). The patient displayed persecutory delusions, irritability, mild depression and apathy. The patient's family described that the patient was afraid that others would steal his things and hurt him, so he installed a camera at home. Besides, he did not care about family and was irritable. His ability of daily living has not been affected. He had never taken any antipsychotic drugs. The SARA score was 8 (gait 3, standing 2, sitting 0, poor diction 0, finger tracking test 1, finger-nose test 0, rapid alternation test 0, heel-knee-shin test 2).

Genetic testing identified two heterozygous variants in the SPG7 gene: c.1150_1151insCTAC (p.G384Afs*13) and c.1496delC (p.Q500Sfs*13). Sanger sequencing showed that his son carried the c.1150_1151insCTAC variant, while his daughter carried the c.1496delC variant (Fig. 2b).

Both affected individuals manifested spastic-ataxia, we first detected spinocerebellar ataxia genes(SCA1, 2, 3, 6, 7, 8, 12, and 17, DRPLA and FRDA) by using PCR fragment analysis in the proband. The results were negative. More genetic analyses were listed in Additional file 2, Table S2. Vitamin and folic acid levels were in the normal range. Autoimmune-related and paraneoplastic antibodies were negative (Additional file 3, Table S3). On the basis of these findings, we clinically diagnosed these two patients with complicated SPG7.

## Discussion and conclusions

Variants of the mitochondrial protein paraplegin cause SPG7 and account for the second most common cause of autosomal-recessive spastic paraplegia [3]. To date, there are at least 77 variants in the causative gene SPG7, including missense, nonsense, splice, shift and exon deletion variants [4], and the phenotype can be either simple or complex. In the complex phenotype, clinical symptoms can be associated with cerebellar ataxia, optic neuropathy and ophthalmoparesis. Cerebellar atrophy detected by cranial MRI is the most common feature in some patients.

There are few Chinese families with SPG7 reported in the literature [5] compared with other ethnicities [4, 6]. Here, we report two sporadic cases of SPG7 in a female and a male who presented with symptoms at the age of 41 years, in agreement with the previous reported of onset age between 15 and 60 years, with a median age of onset of 44 years [4]. The initial symptoms of both patients were ataxia, bilateral lower limb weakness, unstable walking, and easy tripping. These symptoms are difficult to distinguish from those of spinocerebellar ataxia and multiple system atrophy C type, so genetic testing is critical for diagnosis. The second patient, who was followed up 3 years later, developed dysarthria and mild cognitive deficits, mainly impaired verbal memory, object naming and executive function. Cognitive impairment has not been described in Chinese patients with SPG7. Similar impaired cognition in the domains of language, verbal memory and executive function was documented and considered to be associated with cerebellar atrophy and cerebello-cortical network dysregulation in six Italian patients with SPG7 [6]. In addition to cognitive impairment, the patient suffered mild psychosis and emotional disorders, especially persecutory delusions. HSP with persecutory delusions is a rare presentation, and the second patient had no early manifestations of cognitive impairment, psychosis and no trauma during adolescence. We ruled out other possible causes of neuropsychiatric symptoms, such as vitamin B12 deficiency. It can be explained that SPG7 is highly expressed in the Purkinje cells of the cerebellum. When the Purkinje cell density of the cerebellum is reduced, resulting in impairment of cerebellum integration, and the cerebellar-thalamic-cortical-cerebellar (CTCC) circuits are damaged, and delusions occur [7]. However, primary psychosis cannot be completely ruled out, and it is necessary to continue follow-up to observe the development of the patient's symptoms. Furthermore, it has been reported that cognitive impairment of SPG7 is related to not only age but also the degree of clinical or subclinical disease progression [8].

The two patients were finally diagnosed by genetic analysis. In the first patient, a compound exon 1–3 heterozygous deletion variant and a c.1150_1151insCTAC (p.G384Afs*13) frameshift variant were shown to be causative, while in the second patient, a c.1496delC deletion variant in exon 11 was found in addition to ac.1150_1151insCTAC variant. Among these variants, the c.1150_1151insCTAC variant has already been reported in four Chinese patients [5], and this shift code variant may be a hotspot variant in Chinese SPG7 patients, whereas p.Ala510Val is the most common SPG7 variant in the UK population [9]. To date, this is the first report of the exon 1–3 heterozygous deletion in a Chinese patient. We found that the c.1496delC deletion variant has not been reported in the HGMD Pro database, and pathogenicity analysis showed that the variant is likely pathogenic. Thus, this c.1496delC variant is a novel variant, and its function needs to be confirmed in validation studies, which will further complement the SPG7 genetic phenotype spectrum.

We report two sporadic cases of complicated HSP associated with variants in the SPG7 gene, which further expands the genetic and phenotypic spectrum in Han Chinese individuals. Patients with SPG7 may have cognitive impairments and psychosis, displaying specific characteristics, which should be of concern.

## Supplementary Information

**Additional file 1: Table S1. **Neuropsychological profile of the second proband.

**Additional file 2: Table S2.** List of Genetic Analysis.

**Additional file 3: Table S3. **List of antibodies screened for paraneoplastic and autoimmune encephalitis.

## Data Availability

The datasets used or analyzed during the current case reports are available from the corresponding author on reasonable request.

## Abbreviations

SPG7: Hereditary Spastic Paraplegia 7
SARA: Ataxia Rating Scale
MMSE: Mini-mental State Examination
MoCA: Montreal Cognitive Assessment
RAVLT: Rey Auditory Verbal Learning Test
ROCF: Rey-Osterrieth Complex Figure Test
NPI: Neuropsychiatric Inventory Questionnaire
CDR: Clinical Dementia Rating
