# Supplementary material for: A novel compound heterozygous SPG7 variant is associated with progressive spastic ataxia and persecutory delusions found in Chinese patients: two case reports
Source: BMC Neurol. 2022 May 30;22:200. doi: 10.1186/s12883-022-02706-1 (PMC9150360; doi:10.1186/s12883-022-02706-1)
Supplement: Supplementary file 1 — Additional file 1: Table S1. Neuropsychological profile of the second proband. [file 12883_2022_2706_MOESM1_ESM.docx]

**Table S1** Neuropsychological profile of the second proband

| **Overall Cognitive Screening** | **test** | **score** | **Cut off for test** |
| --- | --- | --- | --- |
|  | Mini-mental State Examination(MMSE) | 27 | ≤24 |
|  | Montreal Cognitive Assessment (MoCA) | 22* | ≤25 |
| **Cognitive Domains** | **test** | **score** | **Cut off for test** |
| Verbal Memory | Rey Auditory Verbal Learning Test (RAVLT) short-time memory | 6* | <7 |
|  | Rey Auditory Verbal Learning Test (RAVLT) delayed recall | 3* | <6 |
| Language | Verbal Fluency | 16 | <11 |
|  | Boston Naming | 19* | ≤22 |
| Visuospatial Ability | Rey-Osterrieth Complex Figure Test(copy) | 32 | 34.41±1.63 |
| Visuospatial Memory | Rey-Osterrieth Complex Figure Test(recall) | 13 | 19.42±7.07 |
| Executive function | Stroop Test (time score) | 92s* | 28.5±9.5s |
|  | Stroop Test (error score ) | 1 | >1.0 |
| Attention | Forward digit span | 6 | 7±2 |
|  | Backward digit span | 4 | 5±2 |
| **Behavioral and Psychological Symptoms** | neuropsychiatric inventory questionnaire (NPI) | 6* | >0 |
| **Mild cognitive impairment** | Clinical Dementia Rating(CDR) | 0.5* | 0.5 |

*****value under the cut-off normative data; S=seconds**.**
