# Supplementary material for: A novel compound heterozygous SPG7 variant is associated with progressive spastic ataxia and persecutory delusions found in Chinese patients: two case reports
Source: BMC Neurol. 2022 May 30;22:200. doi: 10.1186/s12883-022-02706-1 (PMC9150360; doi:10.1186/s12883-022-02706-1)
Supplement: Supplementary file 3 — Additional file 3: Table S3. List of antibodies screened for paraneoplastic and autoimmune encephalitis. [file 12883_2022_2706_MOESM3_ESM.docx]

**Table S3** List of antibodies screened for paraneoplastic and autoimmune encephalitis

| Serial number | Autoantibody profile of autoimmune encephalitis | Autoantibody profile of paraneoplastic syndrome | Result |
| --- | --- | --- | --- |
| 1 | NMDAR antibody IgG | anti-Hu antibody IgG | Negative |
| 2 | LGI1 antibody IgG | anti-Yo antibody IgG | Negative |
| 3 | CASPR2 antibody IgG | anti-Ri antibody IgG | Negative |
| 4 | GABA_B_R antibody IgG | anti-CV2(CRMP5) antibody IgG | Negative |
| 5 | AMPAR1 antibody IgG | anti-Amphiphysin antibody IgG | Negative |
| 6 | AMPAR2 antibody IgG | anti-Ma1 antibody IgG | Negative |
| 7 | IgLON5 antibody IgG | anti-Ma2 antibody IgG | Negative |
| 8 | DPPX antibody IgG | anti-SOX1 antibody IgG | Negative |
| 9 | GAD65 antibody IgG | anti-Tr(DNER) antibody IgG | Negative |
| 10 | mGluR5 antibody IgG | anti-Zic4 antibody IgG | Negative |
| 11 | GlyR antibody IgG | anti-GAD65 antibody IgG | Negative |
| 12 | D2R antibody IgG | anti-PKCγ antibody IgG | Negative |
| 13 |  | anti-Recoverin antibody IgG | Negative |
| 14 |  | anti-Titin(MGT30) antibody IgG | Negative |
